# Genetic risk and transdiagnostic traits in anorexia nervosa, obsessive-compulsive disorder, and schizophrenia

**DOI:** 10.1017/S0033291725101839

**Published:** 2025-11-25

**Authors:** Stefana Aicoboaie, Edoardo Pappaianni, Mohamed Abdulkadir, Helena Lucy Davies, Nadia Micali

**Affiliations:** 1 Center for Eating and feeding Disorders Research, Mental Health Center Ballerup, Copenhagen University Hospital – Mental Health Services CPH, Denmark; 2 Institute for Biological Psychiatry, Mental Health Center Sct Hans, Copenhagen University Hospital – Mental Health Services, Copenhagen, Denmark; 3 Department of Public Health, National Centre for Register-Based Research, Aarhus University, Aarhus, Denmark; 4 Great Ormond Street Institute of Child Health, University College London, London, UK

**Keywords:** anorexia nervosa, obsessive compulsive disorder, Philadelphia Neurodevelopmental Cohort, polygenic scores, schizophrenia

## Abstract

**Background:**

Shared genetic risk has been shown across psychiatric disorders. In particular, anorexia nervosa (AN), obsessive-compulsive disorder (OCD), and schizophrenia (SCZ) show shared genetic risk that matches clinical evidence of shared illness and cognitive phenotypes. Given this evidence, we leveraged a large US-based population-based study to determine genetic associations of disorder-specific and shared psychiatric, cognitive, and brain markers and explore whether the latter might be state versus trait markers in eating disorders.

**Methods:**

We used data from the population-based Philadelphia Neurodevelopmental Cohort (*N* = 4,729) and conducted sex-stratified analyses to test for associations between genetic risk for three disorders (AN, OCD, and SCZ) and mental health phenotypes, neurocognitive traits, and cortical features in a non-clinical population. Exploratory analyses on cortical features were run on a subset with neuroimaging data (*N* = 626).

**Results:**

Genetic risk for AN was significantly associated with body image distortion (*p*_FDR_ = 0.02), and body image distortion was significantly related to a reduction in grey matter volume (*p*_FDR_ = 0.05).

**Conclusion:**

Genetic risk for AN associates with AN trait in a non-clinical sample of youth, particularly in females. Whilst genetic risk was not associated with cognitive or cortical markers, the AN phenotype was associated with cortical markers.

## Introduction

Eating disorders (EDs), obsessive-compulsive disorder (OCD), and schizophrenia (SCZ) are serious psychiatric illnesses (American Psychiatric Association, 2013; Keshaviah et al., 2014; Pearlson, 2000; Treasure, Duarte, & Schmidt, 2020; World Health Organization, 2008) that impact an individual’s overall well-being. Epidemiological, clinical, and genetic evidence support an overlap between EDs, OCD, and SCZ (Cederlöf et al., 2015; Cross-Disorder Group of the Psychiatric Genomics Consortium, 2019; Foulon, 2003; Godart, Flament, Perdereau, & Jeammet, 2002; Halmi et al., 2005; Hudson & Jonas, 1984, Hudson, Hiripi, Pope, & Kessler, 2007; Khalil, Hachem, & Richa, 2011; Morylowska-Topolska et al., 2017; Swinbourne & Touyz, 2007; Yilmaz et al., 2020).

Epidemiological observations show that EDs and OCD commonly co-occur in the clinic (Godart et al., 2002; Halmi et al., 2005; Hudson et al., 2007; Swinbourne & Touyz, 2007). It is estimated that between 20–60% of patients with EDs have a lifetime history of OCD (Godart et al., 2003; Halmi et al., 2005; Kaye, Bulik, Thornton, Barbarich, & Masters, 2004) and that between 3 and 13% of patients with OCD have a lifetime history of an ED (du Toit, van Kradenburg, Niehaus, & Stein, 2001; Fireman, Koran, Leventhal, & Jacobson, 2001; LaSalle et al., 2004; D. A. Pinto, Mancebo, Eisen, Pagano, & Rasmussen, 2006; Sallet et al., 2010). Studies have also shown that in both clinical and population samples, SCZ psychotic symptoms and EDs are comorbid (Foulon, 2003; Hudson & Jonas, 1984; Khalil et al., 2011; Morylowska-Topolska et al., 2017; Solmi, Mascarell, Zammit, Kirkbride, & Lewis, 2019a).

EDs, particularly Anorexia Nervosa (AN), OCD, and SCZ, also share phenotypic traits such as perfectionism (Bardone-Cone et al., 2007; Bulik et al., 2003), neuroticism (Cassin & von Ranson, 2005; Lilenfeld, 2011; Samuels et al., 2000), obsessive thoughts and repetitive behaviors (Braun, Sunday, & Halmi, 1994; George Hsu, Kaye, & Weltzin, 1993; Halmi et al., 1991; Serpell, Livingstone, Neiderman, & Lask, 2002). In addition to these shared characteristics, patients with both AN and SCZ often present with cognitive disturbances (Morylowska-Topolska et al., 2017). While this disturbance is generalized to the entire cognitive processes in SCZ, AN patients show impaired reasoning in regard to body image, body weight, and eating behavior (Morylowska-Topolska et al., 2017; Powers, Simpson, & McCormick, 2005). It is thought that the observed maladaptive behaviors in ED, OCD, and SCZ reflect altered brain structure or function. Adult AN patients have been shown to have a reduced total intracranial volume (TIV) (Van den Eynde et al., 2012), grey matter volume (GMV), and an increased cerebrospinal fluid (CSF) volume compared to controls (Titova, Hjorth, Schiöth, & Brooks, 2013). Similarly, reduced GMV and cortical thickness have been identified as hallmarks of SCZ (Howes, Cummings, Chapman, & Shatalina, 2023), while impaired functioning of the frontal cortex is commonly observed in patients with OCD (van den Heuvel et al., 2005) Figure 1 highlights shared and disorder-specific cognitive and illness traits across AN, OCD, and SCZ. It is currently unclear whether the cognitive characteristics observed in AN - that are similar to those seen in OCD and SCZ – are state (i.e. follow the clinical diagnosis: the effect of the illness) or trait (i.e. precede clinical diagnosis) manifestations.

**Figure 1.**
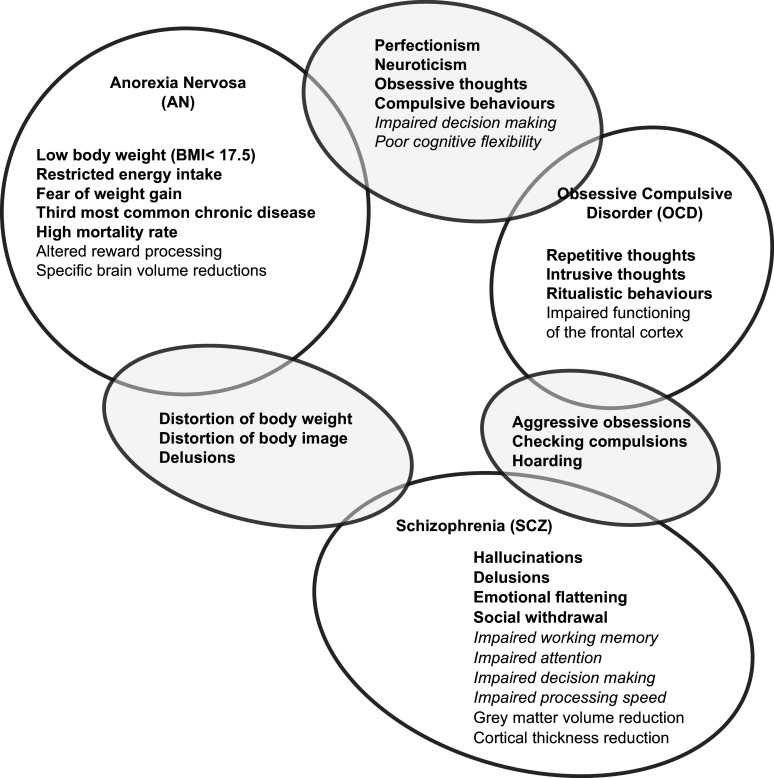
Anorexia nervosa, obsessive compulsive disorder, and schizophrenia characteristics (white circles) and transdiagnostic traits (grey shaded circles). Within each circle, disorder symptoms are identified in bold, cognitive characteristics in italics, and neural characteristics in regular text.

Evidence from genetic studies in the last 5–10 years suggests that these three psychiatric conditions (AN, OCD, SCZ) have a common biological underpinning, suggesting that an overlapping set of genetic markers confers risk for the three disorders. Previous investigations identified the AN-OCD genetic correlation as the 2^nd^ highest among eight analyzed psychiatric conditions (r = 0.50) and reported a moderate genetic correlation between OCD and SCZ (r = 0.35) (Cross-Disorder Group of the Psychiatric Genomics Consortium, 2019). A recent study (Lu et al., 2024) further provided evidence for a genetic overlap between AN and SCZ, identifying 10 genomic loci associated with both disorders. This suggests that genetic risk for one of these psychiatric disorders can increase risk for another.

Polygenic risk scores (PRSs) index an individual’s genetic propensity for a specific disease (Lewis & Vassos, 2020). Results from our group indicate that both PRS for AN and PRS for OCD can inform about AN cognition and behaviours in adolescents in a population-based cohort (Yilmaz et al., 2023). This suggests that genetic risk specific to a given psychiatric disorder can inform about common phenotypic traits.

The literature on AN and psychiatric comorbidities mainly focuses on clinical manifestations of these disorders, disregarding shared symptoms and behaviours across disorders that precede clinical onset (Yilmaz et al., 2023). Moreover, the majority of studies include more severe cases (e.g. clinical studies, case–control studies), which prevents generalization to the general population. However, given that 85% of AN cases are said to begin before the age of 20, with almost all cases starting before the age of 25 (Herpertz-Dahlmann, 2009), it is important to understand how psychiatric traits are expressed in a population at risk (i.e. 8–21 years old) without a clinical diagnosis.

The present study aims to investigate whether polygenic risk for AN, OCD, and SCZ associates with disorder-specific and shared clinical, neurocognitive, and cortical phenotypes in a non-clinical population sample. Given shared genetics between AN, OCD, and SCZ, we hypothesized that PRSs for AN, OCD, and SCZ are associated with clinical, neurocognitive, and cortical characteristics seen in these disorders. Moreover, we hypothesized that disease-specific polygenic risk to be more informative about disease-specific cognitive and clinical phenotypes compared to non-disease-specific PRS. Lastly, in order to identify cortical markers of AN phenotypes, we performed an additional exploratory analysis focusing on the relationship between AN clinical phenotypes and cortical morphometry. Based on previous clinical reports of cortical changes in AN patients (Frank et al., 2015), we expected AN clinical phenotypes to associate with cortical markers. Given the sex differences in psychiatric disorders (Yang et al., 2024), we performed exploratory sex-stratified analyses for all reported investigations. Considering the cross-sectional design of the study, we hereunder use ‘state’ to refer to one-time measurements and ‘trait’ to refer to phenotypes that are genetically determined.

## Methods and materials

### Participants

The Philadelphia Neurodevelopmental Cohort (PNC) is a population-based study of youth living in the greater Philadelphia (United States) area (Calkins et al., 2015) that aims to describe how genetics impacts brain development and cognitive functioning in adolescents (Satterthwaite et al., 2014).

Between 2006 and 2012, 50,293 participants were recruited by the Children’s Hospital of Philadelphia Center for Applied Genomics through a network of non-psychiatric pediatric clinics. To be included in the study, participants had to be between 8 and 21 years old, provide written informed consent to be re-contacted for future studies, be proficient in English, and not have any significant developmental delay or physical conditions that would interfere with study completion (Calkins et al., 2015). Overall, 19,161 individuals met the inclusion criteria; 9,428 (49.2%) of these completed clinical and neurocognitive assessments. Data cleaning was performed only on genotyped individuals (N = 8,139); 650 individuals were removed due to relatedness, and 2,760 individuals were removed due to non-European ancestry (2,050 individuals of African descent and about 700 admixed individuals). A total of 4,729 participants with genotypic data and at least one clinical or neurocognitive measure remained eligible for analysis.

### Measures

#### Clinical measures

All participants underwent a clinical assessment using a computerized structured interview (Kiddie Schedule for Affective Disorders and Schizophrenia (K-SADS) Family Study Interview (Kaufman et al., 1997) that was administered to an informant (caregiver or legal guardian), for those between 8 and 10 years old, to an informant and probands, for those between 11 and 17 years old, and to probands only for those aged 18–21 years old. The interview assessed demographics, medical history, and psychopathology. For our study, we focused on the items assessing AN (2 items), OCD (based on previous studies on this cohort, dimension scores were generated (Bralten et al., 2020), and SCZ (5 items assessing auditory, visual, olfactory, tactile hallucinations and delusional beliefs) (Calkins et al., 2015). Questions about symptom frequency, symptom/episode duration, onset/offset of symptoms/episodes were excluded from the analyses. See Supplementary Table 1 for a list of the K-SADS items included.

#### Cognitive measures

Cognitive performance was assessed using the computerized neurocognitive battery (CNB) (Gur et al., 2010). The CNB assesses accuracy and speed in abstraction and mental flexibility, attention, working memory, episodic memory (e.g. word, face, spatial), language reasoning, spatial processing, sensorimotor, motor speed, and emotion identification (see Gur et al., 2010 for a complete review). The tests cover four cognitive domains (executive control, episodic memory, complex cognition, and social cognition) (Roalf et al., 2013). A standardized reading test (Wide Range Achievement Test, WRAT4 (Wilkinson & Robertson, 2006) was also administered prior to the CNB to test participants’ Intelligence Quotient (IQ) and their ability to complete the full battery.

The neurocognitive scores were computed following previous reports (Moore, Reise, Gur, Hakonarson, & Gur, 2015). Raw accuracy and speed scores were converted to z-scores based on the sample’s mean and standard deviation. Speed values were computed by multiplying by −1 the z-scores for median response time. Accuracy scores and speed values were then summed to create an efficiency score. For example, an individual with an accuracy score of 2.50 (i.e. very accurate) and a speed score of −2.50 (i.e. very slow) had an overall efficiency score of 0. Domain-specific scores were calculated as the average of different efficiency scores. The executive functioning domain score was calculated as the average of three efficiency scores: abstraction, attention, and working memory. The episodic memory domain score was computed as the average of verbal, face, and spatial memory efficiency scores. The complex cognition score reflected the average of language and analogical reasoning, nonverbal reasoning, as well as spatial processing efficiency scores. The social cognition domain score was computed as the average of emotion processing, emotion differentiation, and age differentiation efficiency scores (Supplementary Table 2).

#### Cortical measures

A subsample of participants (N = 1,598) underwent a Magnetic Resonance Imaging (MRI) scan on a 3 T Siemens TIMTrio scanner at the Hospital of the University of Pennsylvania (see Satterthwaite et al., 2014 for all the details regarding the neuroimaging protocol). A sub-analysis was carried out only on participants with complete data (i.e. genetic, clinical, neurocognitive, and neuroimaging data) (n = 627). Of the 627 subjects with complete data, 626 had a Magnetization Prepared Rapid Gradient Echo Imaging (MPRAGE) T1-weighted structural MRI image available, acquired with Repetition Time (TR) = 1810 ms, Echo Time (TE) = 3.5 ms, slice thickness = 1 mm, flip angle = 9°, field-of-view (FoV) = 180 mm (Satterthwaite et al., 2014). The MPRAGE image was used to extract GMV, white matter volume (WMV), CSF, TIV, cortical thickness, and gyrification.

#### Genotype data

The Center for Applied Genomics performed the genotyping of the entire PNC cohort (Satterthwaite et al., 2014) (for a complete overview of the genotyping pipeline, see Glessner et al., 2009; D. Pinto et al., 2010). The genotyping was performed on one of the three Illumina platforms (HumanHap550, HumanHap610, or OmniExpress v2 (Robinson et al., 2015). All data were retrieved through the National Institute of Mental Health’s Database of Genotypes and Phenotypes (dbGaP) (dbGaP, 2014) (PNC).

### Missing data

Given that less than 20% of the data per variable was missing at random (range: 0.7–4.5%), multivariate imputation by chained equations (MICE) (*mice* package in R (Buuren & Groothuis-Oudshoorn, 2011) was used to estimate missing values for the clinical and neurocognitive variables. A prediction matrix was created to set which variables inform the imputation of which variable (Supplementary Table 8). Thus, clinical variables only informed the imputation of other clinical variables, while neurocognitive variables only informed the imputation of other neurocognitive variables. Biological variables such as sex, height, weight, and PRSs were not imputed. No data imputation was run on the neuroimaging dataset. Dichotomous clinical variables were imputed using the Bayesian logistic regression method (Rubin, 1987), while numeric cognitive variables were imputed using the predictive mean matching method. Five imputations were first run on the entire sample as the default setting in the *mice* package. The number of imputations was stepwise increased until the largest fraction of missing information (*fmi*) was 100 times smaller than the number of imputations (Madley-Dowd, Hughes, Tilling, & Heron, 2019). Seven imputations were needed to satisfy this criterion. As a second stage, imputations were run separately on female- and male-only datasets. Eight imputations were required to meet the *fmi* condition. Summary scores (e.g. obsession, compulsion, executive component, episodic memory, complex cognition, and social cognition scores) were created after the imputation of the raw variables.

The quality of the imputation was assessed via diagnostic plots (e.g. plots of the mean and standard deviation of the imputed values against the iteration number).

### Statistical analyses

#### Polygenic risk scores

PRSs were computed using the polygenic risk score continuous shrinkage (PRS-CS) software (Ge, Chen, Ni, Feng, & Smoller, 2019). The discovery sample consisted of previously released GWAS summary statistics (Freeze 2 AN GWAS (Watson et al., 2019), Freeze 1 OCD GWAS (Arnold et al., 2018), and Freeze 3 SCZ GWAS (Schizophrenia Working Group of the Psychiatric Genomics Consortium, 2022) and the target sample consisted of genotyped PNC participants of European ancestry.

#### Neuroimaging

All MPRAGE structural scans were preprocessed using the standard pipeline included in the Computational Anatomy Toolbox v. 12 (CAT12) (https://neuro-jena.github.io/cat/) and the Statistical Parametric Mapping (SPM12) toolbox (https://www.fil.ion.ucl.ac.uk/spm/software) in MATLAB (The Mathworks). Given the age range of our subjects (i.e. 8–21), a customized tissue probability map created with the CerebroMatic toolbox (Wilke, Altaye, Holland, & Consortium, 2017) was used during preprocessing. The toolbox contains a dataset of more than 1000 brain scans of subjects between the ages of 13 months to 75 years old. The customization was done based on participants’ age and sex, as well as the strength of the MRI magnetic field. GMV, WMV, CSF, and TIV were extracted from CAT12 for each participant, while cortical thickness and gyrification values were extracted from each region-of-interest (ROIs) after cortical parcellation based on the Destrieux Atlas (Destrieux, Fischl, Dale, & Halgren, 2010). Cortical thickness and gyrification were averaged across all ROIs to get a mean value per participant.

#### PRS analyses

Regression models with clinical or neurocognitive variables as dependent variables and PRS as independent variables were used to test for association. Logistic regressions were employed for the binary dependent variables (e.g. body image distortion, binge eating (BE), hoarding, and perfectionism) based on genetic risk (i.e. PRS). For the dependent variables derived by summing across different item variables (e.g., obsession scores), negative binomial regressions were employed to account for over-dispersed count data. For continuous dependent variables (i.e., cognitive traits), linear regression models were run. The regression models were run on each imputed dataset. Each dataset had a different value for the imputed data (non-missing values remain unchanged) because of the random component. As a result, the regression parameter estimates slightly differed between the datasets. The results were then combined to account for the variation in parameter estimates.

#### Neuroimaging data analyses

Exploratory analyses were run on a subset of the sample with available neuroimaging data (N = 626, 309 females), Mean age = 14.47(Standard Deviation (SD) = 3.64). Linear regression models with averaged cortical parameters as dependent variables and PRS as independent variables tested the association between PRS and cortical features. Moreover, unadjusted and adjusted (for age and parental socio-economic status (SES), calculated as the mean of parents’ education) linear regression models with AN clinical traits as dichotomous independent variables and cortical parameters as continuous dependent variables were used to model the association between AN traits and cortical characteristics.

Regression models, including PRSs, were corrected for the first four principal components. The analyses were first run on the entire sample and then on the sex-stratified datasets. Each type of analysis (e.g., mental health, neurocognitive, cortical) was corrected for multiple comparisons using the Benjamini–Hochberg procedure (False-Discovery Rate (FDR) (Benjamini & Hochberg, 1995). Given the exploratory nature of our research, we reported both unadjusted and FDR-adjusted analyses. All analyses were run on R version 4.1.1 (R Core Team, 2021). Brain and genetics computations were performed at the University of Geneva using the Baobab HPC service.

## Results

### Socio-demographic characteristics

The mean age of our sample (2343 females and 2373 males) was 13.81 years (Standard Deviation = 3.67; Range = 14). Table 1 describes participant characteristics.

**Table 1.** Demographics and clinical information

Characteristics	Philadelphia neurodevelopmental cohort *N* = 4,729
Sex (F/M)	2,343/2,373
Age in years, mean (SD)	13.81(3.67)
Parental socioeconomic status, mean years of education, (SD)	15.05 (2.22)

### Mental health phenotypes

A higher AN PRS was significantly associated with body image distortion (p_FDR_ = 0.02); a one standard deviation increase in the AN PRS corresponded to 1.21 (95% Confidence intervals [CI] = 1.08, 1.35) increased odds of endorsing body image distortion. Additionally, some results passed the threshold of nominal significance (p_uncorrected_ < = 0.05): a higher AN PRS associated with compulsive behavior (β = 0.10; 95% CI = 0.01, 0.19; p_uncorrected_ = 0.03), a higher SCZ PRS associated with BE (OR = 1.12; 95% CI = 1.01, 1.23; p_uncorrected_ = 0.03) as well as perfectionism (OR = 1.10; 95% CI = 1.02, 1.19; p_uncorrected_ = 0.02). Moreover, the OCD PRS was associated with psychotic traits (β = 0.07; 95% CI = 0.00, 0.15; p_uncorrected_ = 0.05; Table 2).

**Table 2.** Associations between PRSs and mental health traits (*N* = 4,729): Results from logistic/linear regressions

Phenotype	PRS	OR/β (95% CI)	Z	P	P (FDR adjusted)
*AN*					
Body image distortion	AN	1.21 (1.08, 1.35)	3.29	<0.001*	0.02*
Body image distortion	OCD	1.04 (0.94, 1.16)	0.77	0.44	0.64
Body image distortion	SCZ	1.04 (0.93–1.16)	0.65	0.52	0.64
BE	AN	1.09 (0.98, 1.20)	1.64	0.10	0.27
BE	OCD	1.03 (0.94, 1.13)	0.66	0.51	0.64
BE	SCZ	1.12 (1.01, 1.23)	2.19	0.03*	0.18
*OCD*					
Hoarding	AN	1.11 (0.97, 1.28)	1.50	0.13	0.31
Hoarding	OCD	1.04 (0.91, 1.18)	0.56	0.57	0.67
Hoarding	SCZ	1.03 (0.90, 1.19)	0.46	0.61	0.71
Perfectionism	AN	1.08 (1.00, 1.70)	1.90	0.06	0.18
Perfectionism	OCD	1.03 (0.96, 1.10)	0.77	0.44	0.64
Perfectionism	SCZ	1.10 (1.02, 1.19)	2.41	0.02*	0.17
Obsession	AN	0.08 (0.00, 0.16)	1.88	0.06	0.18
Obsession	OCD	0.03 (−0.05, 0.10)	0.71	0.48	0.64
Obsession	SCZ	0.05 (−0.03, 0.13)	1.25	0.21	0.44
Compulsion	AN	0.10 (0.01, 0.19)	2.12	0.03*	0.18
Compulsion	OCD	0.05 (−0.04, 0.14)	1.07	0.28	0.54
Compulsion	SCZ	0.04(−0.05, 0.12)	0.79	0.43	0.64
*SCZ*					
Psychosis	AN	0.01 (−0.07, 0.09)	0.20	0.84	0.84
Psychosis	OCD	0.07 (0.00, 0.15)	2.00	0.05*	0.18
Psychosis	SCZ	−0.01(−0.09, 0.06)	−0.32	0.75	0.79

AN: Anorexia Nervosa; BE: Binge Eating; OCD: Obsessive Compulsive Disorder; SCZ: Schizophrenia; FDR: False-Discovery Rate.

* *p* < = 0.05.

Associations between AN PRS and body image distortion, and SCZ PRS and perfectionism were observed in the female-only dataset (OR = 1.24; 95% CI = 1.08, 1.41; p_FDR_ = 0.04; OR = 1.15; 95% CI = 1.04, 1.28; p_uncorrected_ = 0.01, respectively). The association between AN PRS and perfectionism showed nominal significance amongst females (OR = 1.12; 95% CI = 1.00, 1.25; p_uncorrected_ = 0.04) (Supplementary Table 3), while a nominally significant association between SCZ PRS and BE was seen in males (OR = 1.21; 95%CI =1.04, 1.40; p_uncorrected_ = 0.01) (Supplementary Table 3).

### Cognitive traits

No associations passed the threshold of significance (nominal or corrected for multiple comparisons) regardless of biological sex (Table 3).

**Table 3.** Associations between PRSs and cognitive measures (N = 4729): Results from linear regression

Phenotype	PRS	β (95% CI)	Z	P	P (FDR adjusted)
Executive control	AN	−0.01 (−0.05, 0.02)	−0.71	0.48	0.64
Executive control	OCD	0.00 (−0.03, 0.04)	0.13	0.90	0.90
Executive control	SCZ	−0.02(−0.06, 0.02)	−0.86	0.39	0.59
Episodic memory	AN	−0.01 (−0.05, 0.03)	−0.45	0.65	0.78
Episodic memory	OCD	0.02 (−0.01, 0.06)	1.23	0.22	0.56
Episodic memory	SCZ	– 0.02 (−0.05, 0.02)	−0.89	0.37	0.59
Complex cognition	AN	−0.02 (−0.05, 0.01)	−1.49	0.14	0.56
Complex cognition	OCD	0.02(−0.01, 0.05)	1.20	0.23	0.56
Complex cognition	SCZ	−0.03(−0.06, 0.00)	−1.77	0.08	0.56
Social cognition	AN	−0.02 (−0.06, 0.02)	−1.02	0.31	0.59
Social cognition	OCD	0.01 (−0.03, 0.04)	0.30	0.76	0.83
Social cognition	SCZ	−0.02(−0.06, 0.01)	−1.33	0.18	0.56

AN: Anorexia Nervosa; OCD: Obsessive Compulsive Disorder; PRS: Polygenic Risk Score; SCZ: Schizophrenia.

### Cortical features

There was a nominal significant association between the AN PRS and CSF volume (*β* = 4.43; 95% CI =1.40, 7.47; p_uncorrected_ < 0.01) (Table 4). Trends in tested associations were observed in the females only dataset: OCD PRS and TIV (*β* = −17.08; 95%CI = −30.25, −3.90; p_uncorrected_ = 0.01) and OCD PRS and GMV, respectively (*β =* −8.93; 95%CI = −16.72, −1.15; p_uncorrected_ = 0.03); and in the males only dataset: AN PRS and TIV (*β* = 17.55; 95%CI =2.37, 32.73; p_uncorrected_ = 0.02), AN PRS and CSF (*β* = 5.99; 95% CI =1.65, 10.33; p_uncorrected_ = 0.01) (Supplementary Table 5).

**Table 4.** Associations between PRSs and cortical phenotypes (*N* = 626): Results from linear regression

Phenotype	PRS	β (95% CI)	Z	P	P (FDR adjusted)
TIV	AN	8.75 (−3.56, 21.07)	1.40	0.16	0.90
TIV	OCD	−6.44 (−17.82, 4.95)	−1.11	0.27	0.90
TIV	SCZ	1.31 (−10.13, 12.75)	0.23	0.82	0.90
GM	AN	2.08 (−4.71, 8.86)	0.60	0.55	0.90
GM	OCD	−3.47 (−9.74, 2.79)	−1.09	0.28	0.90
GM	SCZ	−2.10 (−4.19, 8.39)	0.66	0.51	0.90
WM	AN	2.25(−3.43, 7.93)	0.78	0.44	0.90
WM	OCD	−2.53 (−7.78, 2.71)	−0.95	0.34	0.90
WM	SCZ	−1.05 (−6.323, 4.21)	−0.39	0.69	0.90
CSF	AN	4.43 (1.40, 7.47)	2.87	< 0.01*	0.08
CSF	OCD	−0.43 (−3.26, 2.39)	−0.30	0.76	0.90
CSF	SCZ	0.26 (−2.57, 3.10)	0.18	0.85	0.90
Gyrification	AN	0.00 (−0.07, 0.08)	0.09	0.93	0.93
Gyrification	OCD	−0.06 (−0.13, 0.01)	−1.67	0.10	0.86
Gyrification	SCZ	0.03(−0.04, 0.10)	0.86	0.39	0.90
Thickness	AN	0.00 (−0.01, 0.00)	−0.43	0.67	0.90
Thickness	OCD	0.00 (−0.01, 0.01)	0.23	0.82	0.90
Thickness	SCZ	0.00(−0.01, 0.01)	−0.23	0.82	0.90

AN, anorexia nervosa; CSF, cerebrospinal fluid; GMV, grey matter volume; OCD, obsessive compulsive disorder; PRS, polygenic risk score; SCZ, schizophrenia; TIV, total intracranial volume; WMV, white matter volume.

* *p* < = 0.05.

In the exploratory analyses focusing on the associations between ED traits (i.e. body image distortion and BE) and brain measures, there was a significant association between body image distortion and reduced GMV (*β* = −37.56; 95% CI = −60.19, −14.94; p_FDR_ = 0.01) and reduced mean cortical thickness (*β* = −0.05; 95%CI = −0.08, −0.01; p_FDR_ = 0.04). Body image disturbance was nominally associated with reduced TIV (*β* = −47.75; 95%CI = −88.73, −6.77; p_uncorrected_ = 0.02). BE also showed a nominally significant association with reduced mean gyrification (*β* = −0.28; 95%CI = −0.50, −0.06; p_uncorrected_ = 0.01) (Table 5). Only the association between body image distortion and reduced GMV remained significant after adjustment for SES and age (Table 5). Nominal associations between body image disturbance and GMV and BE and cortical gyrification were observed in the female-only dataset (*β* = −23.81; 95%CI = −47.32, −0.29; p_uncorrected_ = 0.05; *β* = −0.37; 95%CI = −0.67, −0.07, p_uncorrected_ = 0.02, respectively). The results remained significant after correction for SES and age (Supplementary Table 6).

**Table 5.** Associations between ED traits and cortical phenotypes (*N* = 626): Results from unadjusted and adjusted^a^ linear regression analyses

		Unadjusted				Adjusted^a^			
Phenotype	ED trait	β (95% CI)	Z	P	P (FDR adjusted)	β (95% CI)	Z	P	P (FDR adjusted)
TIV	Body image distortion	−47.75 (−88.73, −6.77)	−2.29	0.02*	0.07	−41.61 (−82.88, −0.33)	−1.98	0.05*	0.14
TIV	BE	−17.50 (−53.06, 18.06)	−0.97	0.33	0.57	−17.60 (−53.05, 17.87)	−0.98	0.33	0.57
GMV	Body image distortion	−37.56 (−60.19–14.94)	−3.26	<.001*	0.01*	−30.07 (−50.76, −9.38)	−2.86	.004*	0.05*
GMV	BE	−19.41 (−39.09, 0.26)	−1.94	0.05	0.13	−11.87 (−29.69, 5.95)	−1.31	0.19	0.38
WMV	Body image distortion	−8.37 (−27.31, 10.57)	−0.87	0.39	0.58	−8.33 (−26.95, 10.29)	−0.88	0.38	0.57
WMV	BE	−0.82 (−17.20, 15.57)	−0.10	0.92	0.92	−4.57 (−20.53, 11.39)	−0.56	0.57	0.69
CSF	Body image distortion	−1.82 (−12.03, 8.38)	−0.35	0.73	0.87	−3.21 (−12.20, 5.78)	−0.70	0.48	0.64
CSF	BE	2.73 (−6.09, 11.55)	0.61	0.54	0.73	−1.16 (−8.87, 6.54)	−0.30	0.77	0.84
Gyrification	Body image distortion	−0.22 (−0.48, 0.04)	−1.65	0.10	0.20	−0.19 (−0.44, 0.06)	−1.52	0.13	0.31
Gyrification	BE	−0.28 (−0.50, −0.06)	−2.47	0.01*	0.06	−0.22 (−0.43, 0.00)	−1.99	0.05*	0.14
Thickness	Body image distortion	−0.05 (−0.08, −0.01)	−2.71	0.01*	0.04*	−0.04 (−0.08, −0.01)	−2.33	0.02*	0.12
Thickness	BE	0.00 (−0.03, 0.03)	0.19	0.85	0.92	0.01 (−0.02, 0.04)	0.45	0.65	0.71

a Adjusted for age and socioeconomic status.

BE: Binge Eating; CSF: Cerebrospinal Fluid; GMV: Grey Matter Volume; TIV: Total Intracranial Volume; WMV: White Matter Volume.

* *p* < = 0.05.

Amongst males, nominal associations were observed between body image distortion and TIV (*β* = 71.88; 95%CI = 3.67, 140.09; p_uncorrected_ = 0.04) and WMV, respectively (*β* = 43.88; 95%CI = 9.05, 78.72; p_uncorrected_ = 0.01). Only the association between body image distortion and WMV persisted after adjusting for SES and age (Supplementary Table 7).

## Discussion

In line with our hypotheses, in this large population-based study, we observed that PRSs for three psychiatric disorders are associated with clinical and cortical characteristics seen in these disorders. The AN PRS was significantly associated with an AN trait in a non-clinical youth population: those with a high AN PRS were at a higher risk of expressing body image distortion. Interestingly, in our sex-stratified analyses, we observed this association amongst females, suggesting that females and males might differ in their genetic propensity for body image distortion. It is also possible that these associations are largely due to sex imbalances (females: 14,898 cases and 27,545 controls vs males: 447 cases and 20,347 controls) in the original GWAS from which we derived our PRS (Watson et al., 2019). Overall, the findings agree with previous work that showed that AN PRS can significantly inform about AN traits in the general population (Curtis et al., 2024; Yilmaz et al., 2023).

The results also confirmed our second hypothesis that disease-specific PRS can better inform about disease-specific phenotypes than related PRS. For example, AN PRS informed better than any other psychiatric PRS about AN-specific symptomatology (i.e. body image distortion). Nevertheless, we also observed trends in associations that cross diagnostic boundaries. For example, SCZ PRS associated with BE and perfectionism, and AN PRS associated with compulsive behavior. These findings are in line with previous findings (Lundgren, Rempfer, Brown, Goetz, & Hamera, 2010; Ramacciotti et al., 2004; Solmi, Mascarell, Zammit, Kirkbride, & Lewis, 2019b) and add to the literature that supports an association between EDs and SCZ (Bulik-Sullivan et al., 2015; Duncan et al., 2017; Zhang et al., 2021). We observed some sex-specific trends in PRS associations, with the AN and SCZ PRSs showing marginal evidence of an association with perfectionism amongst females and the SCZ PRS nominally associating with BE in males. These cross-disorder associations might result from spurious associations when testing multiple outcomes or might point to higher-order psychopathological factors like factor *p.* While nominally significant, these associations require further validation for a conclusive statement on the transdiagnostic effects of genetic risk.

Our study failed to replicate previous associations between psychiatric PRS and neurocognitive traits (Hatzimanolis et al., 2015; Mallet, Strat, Dubertret, & Gorwood, 2020). One possible explanation for the lack of results is that the cognitive alterations seen in clinical populations, on which the PRSs are computed, are extreme and might not be present at the population level, or they might be state markers of clinical diagnoses. Another possible explanation is that the chosen neurocognitive traits might not be good trait markers for the disorders. One study looking at the most suitable cognitive markers for SCZ identified a measure of sustained attention and a measure of general cognitive function as the best candidates, based on the shared genetic effect with SCZ (McCarthy et al., 2017).

The lack of association between AN PRS and global cortical volume is in line with recent reports showing a nominal association between AN PRS and reduced caudate volume but no associations with global cortical measures (e.g. cortical thickness, surface area) (Westwater et al., 2023). While we did not observe any significant associations between PRSs and cortical features, we did observe a significant association between body image distortion and GMV in the whole group and in the female-only group. The lack of significant associations between the included PRSs and cortical characteristics, in the presence of an association between mental health traits and cortical characteristics (specifically for AN), suggests that brain alterations might be state markers and not trait markers. This again supports previous reports of restoration of cortical thinning to a normal level in AN patients following weight gain, further suggesting that brain alterations follow the clinical diagnosis and are not neural traits of the disorder (Bernardoni et al., 2016; King et al., 2015; Nickel et al., 2018).

Our analysis failed to replicate previous reports of associations between PRS SCZ and impaired cognitive functioning (Germine et al., 2016; Hagenaars et al., 2016; Liebers et al., 2016) and between OCD PRS and brain region functioning (Heinzel et al., 2021). The lack of associations between OCD and SCZ PRS and cognitive or cortical traits can be seen in the light of our methodology, for example, the choice of cognitive measures and focus on structural brain morphometrics. To the best of our knowledge, no other investigation, looked at the association between genetic risk for OCD and SCZ, indexed as PRSs, and global cognitive and cortical measures in a sample of young adults.

### Strengths and limitations

The present study provides a comprehensive view of the relationship between three psychiatric disorders PRSs and several phenotypes and neural characteristics (i.e. clinical, neurocognitive, and neurocortical) in a non-clinical population during an important time window for psychiatric disorder development.

The current study has many methodological strengths that are worth mentioning. First, the approaches employed in the data analysis aimed to maximize data availability (e.g. imputation of missing data). Second, our analyses were data-driven, without *stringent a priori* hypotheses. This allowed the exploration of all the available data, without bias. This approach allowed the expansion of the current knowledge outside the previously tested hypotheses. Additionally, a tailored brain template was used, adequate for our sample (i.e., age and gender), for our neuroimaging analysis. This allowed to capture of cortical changes linked to development that might have otherwise been missed.

Several limitations need to be acknowledged. First, despite relying on the K-SADS, a validated instrument assessing psychiatric disorders, the assessment for ED symptoms was fairly short (i.e., 2 clinical items). Moreover, we were unable to incorporate a measure of body mass index (BMI) into the computation of a variable that can better reflect the AN symptomatology, as we were limited by the availability of anthropometric data (i.e., 20% missing for height and 19% missing for weight). Second, we were limited in our power in the males-only dataset by the unbalance of AN symptoms between females and males (i.e., low prevalence of AN symptoms in males) (Supplementary Table 1). Third, the AN PRS, a measure derived mainly from females with an AN diagnosis (Watson, Diemer, et al., 2019), was tested against male-reported AN symptoms, which might explain our inability to detect any significant associations between AN PRS and AN traits in the male sample. Additionally, while we did try to account for the age heterogeneity in the neuroimaging analyses, we acknowledge that the sample age range spans important developmental time points (pre-teens, teens, and young youth) that are worth investigating in greater detail. Additionally, the cross-sectional design of the study limits our ability to make inferences about state vs trait in the true sense of the word. While we do not have any information on the temporality of behaviours (i.e., when a certain behavior occurs), we assumed that since genetic predisposition remains constant throughout a lifetime that the absence of an association between PRS and any phenotype could suggest that the specific characteristic (cognitive, cortical, or clinical) is a consequence of the illness itself. Finally, we limited our analysis to individuals of European descent in the absence of a genome-wide association study (GWAS) of our traits for individuals of African descent and PRS methods that deal well with admixed individuals. However, we showed in our sensitivity analysis (Supplementary Table 9) that PRS SCZ performed similarly in the two ancestral groups (European and African), suggesting that not including individuals of African descent into our main analysis did not significantly alter the results and their interpretation.

It is likely that our ability to link mental health traits to genetic propensity will be improved by a better understanding of the genetics of AN, OCD, and SCZ that will come with larger, more ancestrally diverse GWAS. To our knowledge, a new GWAS of AN and binge eating disorder, that should advance our understanding of the underlying biology of these disorders, is underway. Future studies should aim to replicate the present findings in larger longitudinal cohorts, as the results of the current work might have been diluted by the heterogeneity introduced by the large age range and by the sample size. Given the observed differences in genetic risk between males and females, future research should consider stratifying the analysis by biological sex. This would address previously expressed concerns that both sex and developmental timing influence the developmental pathways to psychiatric risk (Yilmaz et al., 2023) and that genetic risk for AN might have different effects on cortical measures in men and women (Leehr et al., 2019).

Lastly, future neuroimaging studies investigating the relation between PRS and cortical parameters should use all the available information. Given that the analyzed data originated from a non-clinical population and no *a priori* hypotheses regarding specific brain regions were formulated, our neuroimaging analyses only looked at global and averaged cortical measures such as TIV, cortical thickness, and GMV. However, it is well established that AN patients show brain changes in specific regions involved in eating-related functions such as the insula and the orbitofrontal cortex (Frank, 2015). Further studies should also focus on ROIs to better understand what role they play in these disorders. Both approaches (e.g., brain region specific and whole brain) are needed and complementary as they can advance our understanding of the effect of psychopathology at the macro and micro scale.

In conclusion, our study shows that genetic risk for AN, quantified through a PRS, can inform about AN traits, while genetic risk for AN, OCD, and SCZ did not associate with cognitive or cortical markers. Furthermore, our results suggest that brain alterations (i.e., reduced GMV and reduced cortical thickness) associated with AN traits are likely to be the effect of illness (i.e., state markers) and not an inherent trait (i.e., trait markers). This study sheds light on the shared genetic risk between AN, OCD, and SCZ and potential transdiagnostic traits.

## Supporting information

Aicoboaie et al. supplementary material 1 Aicoboaie et al. supplementary material

Aicoboaie et al. supplementary material 2 Aicoboaie et al. supplementary material

## Supplementary material

The supplementary material for this article can be found at http://doi.org/10.1017/S0033291725101839.
