# Supplementary material for: Genetic risk and transdiagnostic traits in anorexia nervosa, obsessive-compulsive disorder, and schizophrenia
Source: Psychol Med. 2025 Nov 25;55:e360. doi: 10.1017/S0033291725101839 (PMC12671915; doi:10.1017/S0033291725101839)
Supplement: Aicoboaie et al. supplementary material 1 — Aicoboaie et al. supplementary material [file S0033291725101839sup001.docx]

Supplementary Table 1*. Clinical items assessed in PNC (adapted from Kiddie-SADS Family Study Interview, Kaufman et al., 1997)*

|  | Trait | Code | Question | Female Yes  (N, %) | Male Yes  (N, %) |
| --- | --- | --- | --- | --- | --- |
| AN | Body-image distortion | EAT001 | Was there ever a time when you felt really fat or heavy, but other people said that you were too thin? | 267 (11%) | 90 (4%) |
|  | BE | EAT007 | Has there been a time when your eating was out of control – you’d eat a large amount of food in a short period of time and could not stop yourself? | 254 (11%) | 222 (9%) |
| OCD | Obsession | OCD001 | Have you ever been bothered by thoughts that don’t make sense to you, that come over and over again and won’t go away, such as concern with harming others/self? | 112 (5%) | 88 (4%) |
|  |  | OCD002 | Have you ever been bothered by thoughts that don’t make sense to you, that come over and over again and won’t go away, such as pictures of violent things? | 94 (4%) | 70 (3%) |
|  |  | OCD003 | Have you ever been bothered by thoughts that don’t make sense to you, that come over and over again and won’t go away such as thoughts about contamination/germ illness? | 121 (5%) | 105 (5%) |
|  |  | OCD004 | Have you ever been bothered by thoughts that don’t make sense to you, that come over and over again and won’t go away, such as fear that you would do something/say something bad without intending to? | 190 (8%) | 167 (7%) |
|  |  | OCD005 | Have you ever been bothered by thoughts that don’t make sense to you, that come over and over again and won’t go away, such as feelings that bad things that happened were your fault? | 259 (12%) | 170 (7%) |
|  |  | OCD006 | Have you ever been bothered by thoughts that don’t make sense to you, that come over and over again and won’t go away, such as forbidden/bad thoughts? | 78 (3%) | 78 (3%) |
|  |  | OCD007 | Have you ever been bothered by thoughts that don’t make sense to you, that come over and over again and won’t go away, such as need for symmetry/exactness? | 200 (9%) | 186 (8%) |
|  |  | OCD008 | Have you ever been bothered by thoughts that don’t make sense to you, that come over and over again and won’t go away, such as religious thoughts? | 58 (3%) | 62 (3%) |
|  | Compulsion | OCD011 | Have you ever had to do something over and over again - that would have made you feel really nervous if you couldn't do it, like: cleaning or washing (for example, your hands, house)? | 121 (5%) | 92 (4%) |
|  |  | OCD012 | Have you ever had to do something over and over again - that would have made you feel really nervous if you couldn't do it, like: counting? | 114 (5%) | 72 (3%) |
|  |  | OCD013 | Have you ever had to do something over and over again - that would have made you feel really nervous if you couldn't do it, like: checking (for example, doors, locks, ovens)? | 216 (10%) | 149 (7%) |
|  |  | OCD014 | Have you ever had to do something over and over again - that would have made you feel really nervous if you couldn't do it, like: getting dressed over and over again? | 57 (3%) | 24 (1%) |
|  |  | OCD015 | Have you ever had to do something over and over again - that would have made you feel really nervous if you couldn't do it, like: going in and out a door over and over again? | 31 (1%) | 20 (1%) |
|  |  | OCD016 | Have you ever had to do something over and over again - that would have made you feel really nervous if you couldn't do it, like: ordering or arranging things? | 184 (8%) | 116 (5%) |
|  |  | OCD017 | Have you ever had to do something over and over again - that would have made you feel really nervous if you couldn't do it, like: doing things over and over again at bedtime, like arranging the pillows, sheets, or other things? | 116 (5%) | 85 (4%) |
|  | Hoarding | OCD018 | Have you ever saved up so many things that people complained or they got in the way? | 124 (5%) | 104 (4%) |
|  | Perfectionism | OCD019 | Do you feel the need to do things just right (like they have to be perfect)? | 461 (20%) | 372 (16%) |
| SCZ | Psychosis | PSY001 | Have you ever heard voices when no one was there? | 139 (6 %) | 144 (6%) |
|  |  | PSY029 | Have you ever seen visions or seen things which other people could not see? | 142 (6%) | 113 (5%) |
|  |  | PSY050 | Have you ever smelled strange odours other people could not smell? | 77 (3%) | 61 (3%) |
|  |  | PSY060 | Have you ever had strange feelings in your body like things were crawling on you or someone touching you and nothing or no one was there? | 251 (11%) | 203 (9%) |
|  |  | PSY070 | Have you ever believed in things that most other people or your parents don’t believe in? | 147 (6%) | 122 (5%) |

Supplementary Table 2. *Overview of the computerised neurocognitive battery (readapted from Moore et al., 2014)*

| Cognitive function | Domain | Test |
| --- | --- | --- |
| Executive Control | Mental Flexibility | Penn Conditional Exclusion Test (PCET) |
|  | Attention | Penn Continuous Performance Test (PCPT) |
|  | Working Memory | Letter N-Back (LNB) task |
|  | Verbal Memory | Penn Word Memory task (PWMT) |
| Episodic Memory | Face Memory | Penn Face Memory Task (PFMT) |
|  | Spatial Memory | Visual Object Learning Test (VOLT) |
|  | Language Reasoning | Penn Verbal Reasoning Test (PVRT) |
| Complex Cognition | Nonverbal Reasoning | Penn Matrix Reasoning Test (PMAT) |
|  | Spatial Ability | Penn Line Orientation Test (PLOT) |
| Social Cognition | Emotion Identification | Penn Emotion Identification test (PEIT) |
|  | Emotion Differentiation | Penn Emotion Differentiation Test (PEDT) |
|  | Age Differentiation | Penn Age Differentiation Test (PADT) |

Supplementary Table 3*. Sex-stratified* *associations between PRS and mental health traits:* *Results from linear regression*

|  | **PRS** | **Females (N=2,343)** | | | | **Males (N=2,373)** | | | |
| --- | --- | --- | --- | --- | --- | --- | --- | --- | --- |
| **Phenotype** | **Fem** | **OR/β(95%CI)** | **Z** | **P** | **P (FDR adjusted)** | **OR/β(95%CI)** | **Z** | **P** | **P (FDR adjusted)** |
| AN |  |  |  |  |  |  |  |  |  |
| Body image distortion | AN | 1.24 (1.08, 1.41) | 3.09 | <0.001* | 0.04* | 1.15 (0.91, 1.44) | 1.18 | 0.24 | 0.79 |
| Body image distortion | OCD | 1.03 (0.90, 1.17) | 0.42 | 0.67 | 0.94 | 1.02 (0.83, 1.26) | 0.22 | 0.82 | 0.98 |
| Body image distortion | SCZ | 1.01(0.89-1,15) | -0.16 | 0.87 | 0.99 | 1.13 (0.90,1.42) | 1.03 | 0.30 | 0.80 |
| BE | AN | 1.07 (0.93, 1.23) | 0.97 | 0.33 | 0.69 | 1.12 (0.97, 1.29) | 1.48 | 0.14 | 0.80 |
| BE | OCD | 1.05 (0.92, 1.19) | 0.70 | 0.48 | 0.78 | 1.10 (0.88, 1.16) | 0.10 | 0.92 | 0.98 |
| BE | SCZ | 1.05(0.92-1.20) | 0.72 | 0.47 | 0.78 | 1.21 (1.04,1.40) | 2.50 | 0.01* | 0.26 |
| OCD |  |  |  |  |  |  |  |  |  |
| Hoarding | AN | 1.12 (0.93, 1.35) | 1.17 | 0.24 | 0.69 | 1.12 (0.91, 1.38) | 1.09 | 0.28 | 0.80 |
| Hoarding | OCD | 0.99 (0.82, 1.18) | -0.13 | 0.89 | 0.99 | 1.09 (0.90, 1.32) | 0.87 | 0.39 | 0.84 |
| Hoarding | SCZ | 0.99(0.83-1.20) | -0.02 | 0.99 | 0.99 | 1.07 (0.87-1.32) | 0.65 | 0.52 | 0.90 |
| Perfectionism | AN | 1.12 (1.00, 1.25) | 2.04 | 0.04* | 0.29 | 1.03 (0.92, 1.16) | 0.50 | 0.61 | 0.91 |
| Perfectionism | OCD | 1.06 (0.95, 1.17) | 1.05 | 0.29 | 0.69 | 0.99 (0.89, 1.11) | -0.13 | 0.90 | 0.98 |
| Perfectionism | SCZ | 1.15(1.04,1.28) | 2.66 | 0.01* | 0.08 | 1.03 (0.91,1.15) | 0.45 | 0.65 | 0.91 |
| Obsession | AN | 0.09 (-0.02, 0.20) | 1.67 | 0.09 | 0.40 | 0.05 (-0.07, 0.18) | 0.84 | 0.40 | 0.84 |
| Obsession | OCD | 0.03 (-0.08, 0.13) | 0.48 | 0.64 | 0.94 | 0.00 (-0.11, 0.12) | 0.03 | 0.98 | 0.98 |
| Obsession | SCZ | 0.01(-0.09,0.11) | 0.28 | 0.78 | 0.96 | 0.08 (-0.04 ,0.2) | 1.35 | 0.18 | 0.80 |
| Compulsion | AN | 0.11 (-0.02, 0.23) | 1.67 | 0.10 | 0.40 | 0.09 (-0-05, 0.23) | 1.25 | 0.21 | 0.80 |
| Compulsion | OCD | 0.04 (-0.07, 0.16) | 0.72 | 0.47 | 0.78 | 0.04 (-0.09, 0.17) | 0.65 | 0.52 | 0.90 |
| Compulsion | SCZ | 0.06 (-0.06,0.18) | 1.02 | 0.31 | 0.69 | 0.00 (-0.14, 0.13) | -0.41 | 0.97 | 0.98 |
| SCZ |  |  |  |  |  |  |  |  |  |
| Psychosis | AN | 0.98 (0.88, 1.10) | -0.32 | 0.75 | 0.96 | 1.03 (0.92, 1.16) | 0.54 | 0.59 | 0.91 |
| Psychosis | OCD | 0.07 (-0.04, 0.17) | 1.28 | 0.20 | 0.69 | 0.08 (-0.03, 0.19) | 1.51 | 0.13 | 0.80 |
| Psychosis | SCZ | 0.00 (-0.1,0.1) | 0.00 | 0.99 | 0.99 | -0.02 (-0.14, 0.09) | -0.37 | 0.71 | 0.93 |

AN: Anorexia Nervosa; BE: Binge Eating; OCD: Obsessive Compulsive Disorder; SCZ: Schizophrenia; PRS: Polygenic Risk Score. *: p<= 0.05.

Supplementary Table 4*. Sex-stratified* *associations between PRS and neurocognitive measures:* *Results from linear regression*

|  |  | **Females (N=2,343)** | | | | **Males (N=2,373)** | | | |
| --- | --- | --- | --- | --- | --- | --- | --- | --- | --- |
| **Phenotype** |  | **β(95%CI)** | **Z** | **P** | **P (FDR adjusted)** | **β(95%CI)** | **Z** | **P** | **P (FDR adjusted)** |
| Executive Control | AN  **PRS** | 0.01 (-0.05, 0.06) | 0.30 | 0.77 | 0.91 | -0.03 (-0.09, 0.02) | -1.25 | 0.21 | 0.63 |
| Executive Control | OCD | 0.01 (-0.04, 0.06) | 0.51 | 0.61 | 0.91 | -0.01 (-0.06, 0.04) | -0.47 | 0.64 | 0.76 |
| Executive Control | SCZ | 0.00 (-0.06, 0.04) | -0.28 | 0.78 | 0.91 | -0.02 (-0.07, 0.023) | -0.81 | 0.42 | 0.71 |
| Episodic Memory | AN | 0.02 (-0.04, 0.07) | 0.55 | 0.58 | 0.91 | -0.03 (-0.09, 0.02) | -1.28 | 0.20 | 0.63 |
| Episodic Memory | OCD | 0.02 (-0.03, 0.07) | 0.75 | 0.46 | 0.91 | 0.02 (-0.03, 0.07) | 0.73 | 0.47 | 0.71 |
| Episodic Memory | SCZ | -0.01 (-0.06, 0.05) | -0.34 | 0.74 | 0.96 | -0.02 (-0.07, 0.03) | -0.96 | 0.34 | 0.71 |
| Complex Cognition | AN | 0.00 (-0.04, 0.05) | 0.05 | 0.96 | 0.91 | -0.05 (-0.09, 0.00) | -2.05 | 0.04 | 0.49 |
| Complex Cognition | OCD | 0.02 (-0.02, 0.07) | 1.11 | 0.27 | 0.91 | 0.01 (-0.03, 0.05) | 0.50 | 0.62 | 0.76 |
| Complex Cognition | SCZ | -0.02 (-0.06, 0.02) | -0.94 | 0.34 | 0.91 | -0.03 (-0.07, 0.01) | -1.42 | 0.15 | 0.63 |
| Social Cognition | AN | -0.03 (-0.09, 0.02) | -1.20 | 0.23 | 0.91 | -0.01 (-0.06, 0.04) | -0.39 | 0.69 | 0.76 |
| Social Cognition | OCD | 0.01 (-0.05, 0.06) | 0.21 | 0.83 | 0.91 | -0.01 (-0.06, 0.04) | -0.26 | 0.79 | 0.79 |
| Social Cognition | SCZ | -0.03 (-0.08, 0.02) | -1.18 | 0.23 | 0.91 | -0.02 (-0.07, 0.03) | -0.71 | 0.48 | 0.71 |

PRS: Polygenic Risk Score; AN: Anorexia Nervosa; OCD: Obsessive Compulsive Disorder; SCZ: Schizophrenia.

Supplementary Table 5. *Sex-stratified* *associations between PRS and cortical phenotypes: Results from linear regression*

|  |  | **Female (N=309)** | | | | **Male (N=317)** | | | |
| --- | --- | --- | --- | --- | --- | --- | --- | --- | --- |
| **Phenotype** | **PRS** | **β(95%CI)** | **Z** | **P** | **P (FDR adjusted)** | **β(95%CI)** | **Z** | **P** | **P (FDR adjusted)** |
| TIV | AN | -0.23 (-13.93, 13.47) | -0.03 | 0.97 | 0.97 | 17.55 (2.37, 32.73) | 2.27 | 0.02* | 0.21 |
| TIV | OCD | -17.08 (-30.25, -3.90) | -2.55 | 0.01* | 0.20 | 6.36 (-7.09, 19.80) | 0.93 | 0.35 | 0.63 |
| TIV | SCZ | -1.62 (-15.24, 12.00) | -0.23 | 0.81 | 0.91 | 6.78 (-6.39, 19.96) | 1.01 | 0.31 | 0.62 |
| GMV | AN | -1.82 (-9.90, 6.26) | -0.44 | 0.66 | 0.85 | 5.91 (-2.11, 13.92) | 1.45 | 0.15 | 0.54 |
| GMV | OCD | -8.93 (-16.72, -1.15) | -2.26 | 0.03* | 0.22 | 2.76 (-4.31, 9.82) | 0.77 | 0.44 | 0.75 |
| GMV | SCZ | 0.65 (-7.38, 8.68) | 0.16 | 0.87 | 0.92 | 4.69 (-2.22, 11.60) | 1.33 | 0.18 | 0.53 |
| WMV | AN | -1.41 (-7.74, 4.92) | -0.44 | 0.66 | 0.85 | 5.65 (-2.18, 13.48) | 1.42 | 0.16 | 0.54 |
| WMV | OCD | -5.78 (-11.90, 0.34) | -1.86 | 0.06 | 0.38 | 1.44 (-5.46, 8.35) | 0.41 | 0.68 | 0.82 |
| WMV | SCZ | -3.07 (-9.36, 3.21) | -096 | 0.34 | 0.78 | 1.82 (-4.95, 8.59) | 0.53 | 0.60 | 0.82 |
| CSF | AN | 3.00 (-1.17, 7.17) | 1.42 | 0.16 | 0.57 | 5.99(1.65, 10.33) | 2.71 | 0.01* | 0.13 |
| CSF | OCD | -2.37 (-6.42, 1.69) | -1.15 | 0.25 | 0.75 | 2.16 (-1.70, 6.01) | 1.10 | 0.27 | 0.61 |
| CSF | SCZ | 0.80 (-3.35, 4.95) | 0.38 | 0.70 | 0.84 | 0.27 (-3.51, 4.06) | 0.12 | 0.89 | 0.89 |
| gyrification | AN | 0.03 (-0.08, 0.13) | 0.54 | 0.59 | 0.84 | -0.02 (-0.14, 0.09) | -0.40 | 0.69 | 0.82 |
| gyrification | OCD | -0.04 (-0.14, 0.07) | -0.72 | 0.48 | 0.78 | -0.08 (-0.18, 0.02) | -1.59 | 0.11 | 0.54 |
| gyrification | SCZ | 0.09 (-0.02, 0.19) | 1.62 | 0.11 | 0.48 | -0.02 (-0.12, 0.08) | -0.34 | 0.73 | 0.82 |
| thickness | AN | -0.01 (-0.02, 0.01) | -0.85 | 0.40 | 0.78 | 0.00 (-0.01, 0.02) | 0.27 | 0.78 | 0.83 |
| thickness | OCD | -0.01 (-0.02, 0.01) | -0.81 | 0.42 | 0.78 | 0.01 (-0.01, 0.02) | 1.26 | 0.21 | 0.54 |
| thickness | SCZ | -0.01 (-0.02, 0.01) | -0.76 | 0.44 | 0.78 | 0.00 (-0.01, 0.02) | 0.49 | 0.62 | 0.82 |

TIV: Total Intracranial Volume; GMV: Grey Matter Volume; WMV: White Matter Volume; CSF: Cerebrospinal Fluid; PRS: Polygenic Risk Score; AN: Anorexia Nervosa; OCD: Obsessive Compulsive Disorder; SCZ: Schizophrenia. *: p<= 0.05.

Supplementary Table 6*. Associations between ED traits and cortical phenotypes in females (N=309): Results from unadjusted and adjusted^1^ linear regression analyses*

|  |  | **Unadjusted** | | | | **Adjusted** | | | |
| --- | --- | --- | --- | --- | --- | --- | --- | --- | --- |
| **Phenotype** | **ED trait** | **β(95%CI)** | **Z** | **P** | **P (FDR adjusted)** | **β(95%CI)** | **Z** | **P** | **P (FDR adjusted)** |
| TIV | Body image distortion | -32.42 (-71.91, 7.70) | -1.62 | 0.11 | 0.43 | -27.17 (-66.84, 12.50) | -1.35 | 0.18 | 0.53 |
| TIV | BE | -7.63 (-46.78, 31.51) | -0.39 | 0.70 | 0.77 | -8.81 (-47.61, 30.01) | -0.45 | 0.66 | 0.79 |
| GMV | Body image distortion | -23.81 (-47.32, -0.29) | -1.99 | 0.05* | 0.28 | -21.36 (-41.22, -1.50) | -2.12 | 0.04* | 0.21 |
| GMV | BE | -10.45 (-33.79, 12.89) | -0.88 | 0.38 | 0.71 | -6.32 (-25.83, 13.18) | -0.64 | 0.52 | 0.70 |
| WMV | Body image distortion | -3.66 (-21.83, 14.51) | -0.40 | 0.69 | 0.77 | -1.64 (-19.811, 16.54) | -0.18 | 0.86 | 0.88 |
| WMV | BE | 3.46 (-14.49, 21.40) | 0.38 | 0.71 | 0.77 | 1.36 (-16.37, 19.10) | 0.15 | 0.88 | 0.88 |
| CSF | Body image distortion | -4.95 (-17.00, 7.07) | -0.81 | 0.42 | 0.71 | -4.17 (-14.83, 6.49) | -0.77 | 0.44 | 0.70 |
| CSF | BE | -0.64 (-12.52, 11.25) | -0.11 | 0.92 | 0.92 | -3.84 (-14.25, 6.56) | -0.73 | 0.47 | 0.70 |
| gyrification | Body image distortion | -0.18 (-0.49, 0.12) | -1.19 | 0.24 | 0.57 | -0.22 (-0.51, 0.07) | -1.46 | 0.14 | 0.53 |
| gyrification | BE | -0.37 (-0.67, -0.07) | -2.45 | 0.02* | 0.18 | -0.31 (-0.59, -0.03) | -2.17 | 0.03* | 0.21 |
| thickness | Body image distortion | -0.03 (-0.07, 0.01) | -1.47 | 0.14 | 0.43 | -0.03 (-0.07, 0.02) | -1.23 | 0.22 | 0.53 |
| thickness | BE | 0.01 (-0.03, 0.05) | 0.72 | 0.47 | 0.71 | 0.02 (-0.02, 0.05) | 0.74 | 0.46 | 0.70 |

*^1^*: adjusted for age and socioeconomic status

TIV: Total Intracranial Volume; GMV: Grey Matter Volume; WMV: White Matter Volume; CSF: Cerebrospinal Fluid; BE: Binge Eating.

*p <= 0.05

Supplementary Table 7. *Associations between ED traits and cortical phenotypes in males (N=317): Results from unadjusted and adjusted^1^ linear regression analyses*

|  |  | **Unadjusted** | | | | **Adjusted** | | | |
| --- | --- | --- | --- | --- | --- | --- | --- | --- | --- |
| **Phenotype** | **ED trait** | **β(95%CI)** | **Z** | **P** | **P (FDR adjusted)** | **β(95%CI)** | **Z** | **P** | **P (FDR adjusted)** |
| TIV | Body image distortion | 71.88 (3.67, 140.09) | 2.07 | 0.04* | 0.23 | 65.98 (-1.61, 133.57) | 1.92 | 0.06 | 0.33 |
| TIV | BE | -3.59 (-48.63, 41.44) | -0.16 | 0.88 | 0.88 | -7.11 (-51.78, 37.56) | -0.31 | 0.75 | 0.82 |
| GMV | Body image distortion | 9.09 (-27.49, 45.66) | 0.49 | 0.63 | 0.83 | 20.99 (-12.87, 54.87) | 1.22 | 0.22 | 0.54 |
| GMV | BE | -16.73 (-40.67, 7.19) | -1.38 | 0.17 | 0.36 | -7.94 (-30.24, 14.35) | -0.70 | 0.48 | 0.82 |
| WMV | Body image distortion | 43.88 (9.05, 78.72) | 2.48 | 0.01* | 0.17 | 33.08(0.98, 65.18) | 2.03 | 0.04* | 0.33 |
| WMV | BE | 4.28 ( -18.78, 27.34) | 0.37 | 0.72 | 0.86 | -3.50 (-24.73, 17.73) | -0.32 | 0.75 | 0.82 |
| CSF | Body image distortion | 18.91 (-0.75, 38.57) | 1.89 | 0.06 | 0.24 | 11.90 (-4.97, 28.77) | 1.39 | 0.17 | 0.54 |
| CSF | BE | 8.86 (-4.07, 21.78) | 1.35 | 0.18 | 0.36 | 4.34 (-6.77, 15.44) | 0.76 | 0.44 | 0.82 |
| gyrification | Body image distortion | -0.29 (-0.81, 0.23) | -1.09 | 0.28 | 0.47 | -0.15 (-0.64, 0.34) | -0.59 | 0.55 | 0.82 |
| gyrification | BE | -0.16 (-0.50, 0.18) | -0.95 | 0.34 | 0.52 | -0.06 (-0.38, 0.26) | -0.37 | 0.71 | 0.82 |
| thickness | Body image distortion | -0.05 (-0.12, 0.02) | -1.51 | 0.13 | 0.36 | -0.04 (-0.11, 0.02) | -1.27 | 0.20 | 0.54 |
| thickness | BE | 0.00 (-0.05, 0.04) | -0.15 | 0.88 | 0.88 | 0.00 (-0.04, 0.05) | 0.17 | 0.86 | 0.86 |

*^1^*: adjusted for age and socioeconomic status

TIV: Total Intracranial Volume; GMV: Grey Matter Volume; WMV: White Matter Volume; CSF: Cerebrospinal Fluid; BE: Binge Eating.

*p <= 0.05

Supplementary Table 9. *Associations between PRS SCZ and psychotic like behaviours in the European and African group: Results from logistic regression analyses*

|  |  | European descent (N=4729) | | | | African descent (N=2050) | | | |
| --- | --- | --- | --- | --- | --- | --- | --- | --- | --- |
| **Phenotype** | **PRS** | **Phenotype prevalence**  **(N, %)** | **OR(95%CI)** | **P** | **P**  **(FDR adjusted)** | **Phenotype prevalence**  **(N, %)** | **OR(95%CI)** | **P** | **P**  **(FDR adjusted)** |
| PSY001 | SCZ | 284 (6%) | 0.97 (0.86, 1.09) | 0.58 | 0.74 | 224 (11%) | 0.95 (0.83, 1.09) | 0.47 | 0.77 |
| PSY029 | SCZ | 256 (6%) | 0.92 (0.81, 1.04) | 0.17 | 0.74 | 188 (10%) | 0.86 (0.75, 1.00) | 0.05* | 0.24 |
| PSY050 | SCZ | 139 (3%) | 0.96 (0.81, 1.13) | 0.59 | 0.74 | 132 (7%) | 1.05 (0.88, 1.26) | 0.58 | 0.77 |
| PSY060 | SCZ | 456 (10%) | 1.01 (0.92, 1.12) | 0.81 | 0.81 | 320 (17%) | 0.97 (0.86, 1.09) | 0.61 | 0.77 |
| PSY070 | SCZ | 270 (6%) | 1.04 (0.92, 1.18) | 0.52 | 0.74 | 136 (7%) | 0.98 (0.83, 1.17) | 0.84 | 0.84 |

PRS: Polygenic Risk Score; SCZ: Schizophrenia.
